# Mutational landscape and its clinical significance in paroxysmal nocturnal hemoglobinuria

**DOI:** 10.1038/s41408-021-00451-1

**Published:** 2021-03-16

**Authors:** Fangfei Chen, Shimin Hu, Jing Ruan, Miao Chen, Bing Han

**Affiliations:** 1 Department of Hematology, Peking Union Medical College Hospital, Peking Union Medical College and Chinese Academy of Medical Sciences, Beijing, China; 2 Division of Gastroenterology, The First Affiliated Hospital, Sun Yat-sen University, Guangzhou, China; 3 Department of Hematopathology, The University of Texas MD Anderson Cancer Center, Houston, TX USA

**Keywords:** Disease genetics, Anaemia, Genetics research, Disease genetics

## Dear Editor,

Paroxysmal nocturnal hemoglobinuria (PNH) is an acquired clonal hematopoietic stem cell disorder caused by mutation of the X-linked *PIGA* gene, resulting in a deficient expression of glycosylphosphatidylinositol-anchored proteins, such as CD55 and CD59^1^. Patients with PNH may present with hemolytic anemia, thrombosis, and bone marrow failure. The loss of CD55 and CD59 renders PNH erythrocytes susceptible to intravascular hemolysis and thrombosis.

There is a close relationship between PNH and aplastic anemia (AA). The clinical picture may shift from one to the other during the course of disease^2^. Genes commonly mutated in myeloid neoplasms have been tested in patients with AA, and some carry prognostic significance^3^. For instance, mutations in *PIGA*, *BCOR*, and *BCORL1* correlate with a better response to immunosuppressive therapy and a longer duration of overall survival and progression-free survival in patients with AA, whereas mutations in *DNMT3A, RUNX1*, *JAK2*, *JAK3*, and *CSMD1* are associated with a worse prognosis. However, studies on the mutations of myeloid cancer-related genes in PNH and on the mechanism of PNH clonal expansion are limited or inconclusive^4,5^. On the other hand, thrombosis is the most common complication in patients with PNH^6,7^. Although the risk of thrombosis correlates with the PNH clone size, thrombotic events do occur in patients with small PNH clones. Recent studies have uncovered that mutations rather than *PIGA* may function as additional risk factors for thrombosis, but the results vary among different studies^8,9^.

In this study, we investigated the mutational profiles of 41 patients with newly diagnosed PNH as well as the CD59+ and CD59- cell fractions of peripheral blood from 6 PNH patients by whole-exome sequencing. We further examined the relations between these mutations and patients’ clinical and laboratory parameters, in particular, we examined the roles of these mutations in the expansion of PNH clones and thrombosis.

The study cohort included 12 patients with PNH and 29 with PNH/AA. There were 29 men and 12 women with a median age of 35 years (range, 15–72). Thirty-nine patients had anemia (Hgb median 78 g/L, range 36-140 g/L), 20 had leukopenia (WBC median 4.11 × 10^9^/L, range 1.5–10.9 × 10^9^/L), and 20 had thrombocytopenia (PLT median 110 × 10^9^/L, range 11–349 × 10^9^/L). Fifteen patients had pancytopenia. Twenty-three patients had increased unconjugated bilirubin (UCB) and 39 had increased lactate dehydrogenase (LDH). The median PNH clone sizes were 83% (range, 10–98%), 82% (range, 10–98%), and 48% (range, 0–97%) by the proportions of FLAER- granulocytes, CD59- granulocytes, and CD59- RBCs, respectively.

Thirteen (31.7%) patients had a history of thrombosis. The median PNH clone size (FLAER negative granulocytes) was 83% (range, 18–95%) in those with thrombosis and 83% (range, 10–98%) in those without (p = 0.688). Coronary artery was the most common site of thrombosis (23.1% of events), followed by visceral vein and/or deep vein (15.4% of events each). Four patients had thrombosis in multiple sites. Common inherited hypercoagulable states (factor V Leiden mutation, prothrombin gene mutation, deficiency of protein S, protein C, or antithrombin) were not detected in those PNH patients. There was no difference in the baseline characters between patients with or without thrombosis, except the value of D-dimer (Supplementary Table S1).

**Fig. 1:**
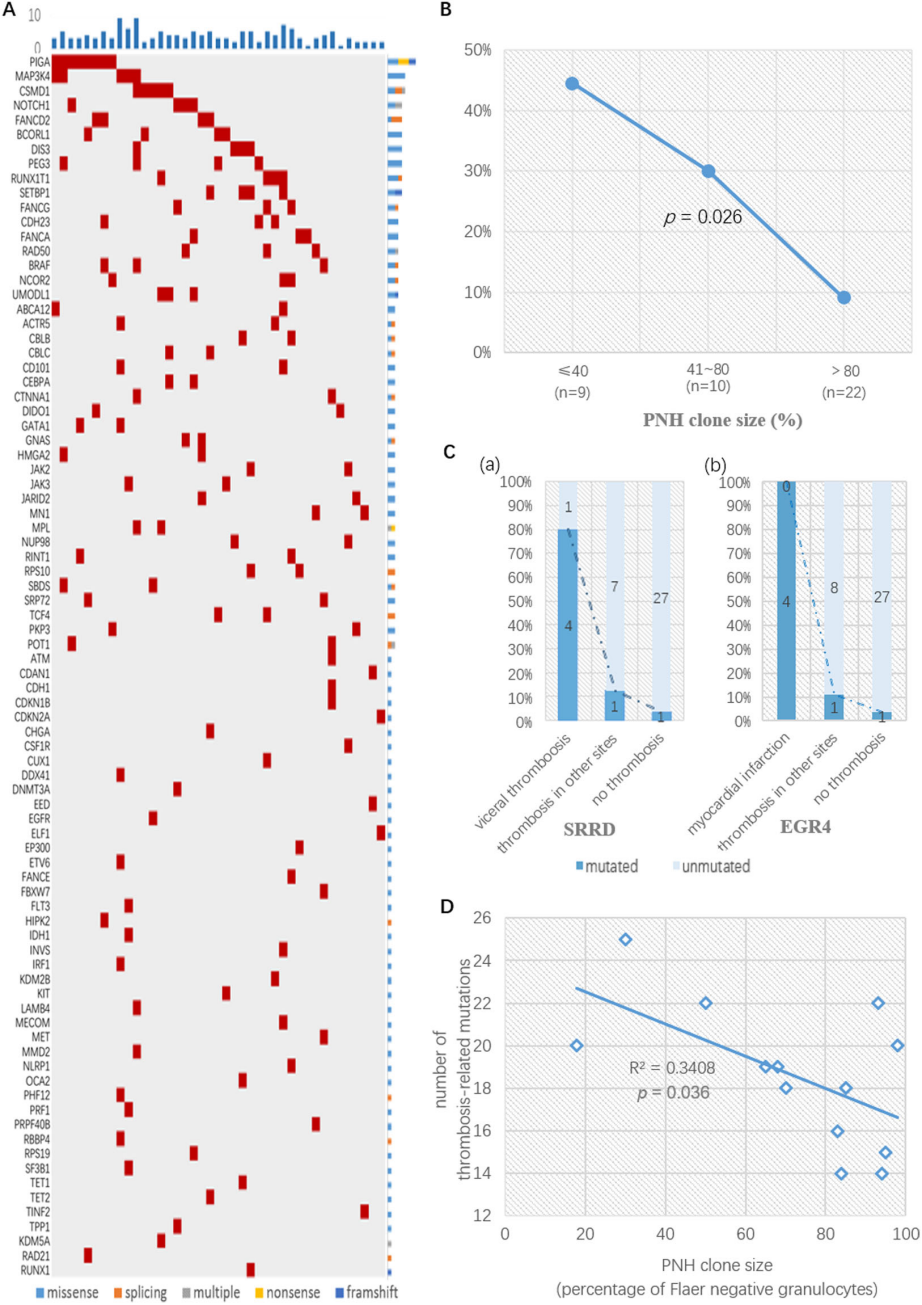
Mutations in PNH and their clinical correlations. **A** Heatmap of mutations in myeloid cancer-related genes detected in PNH. One hundred seventy-eight myeloid cancer-related genes were tested by whole-exome sequencing. Every column represented a patient, and every row represented a mutated gene. Red color indicated the detection of mutated gene in this patient. The bar graph above the heatmap showed the number of mutations in each patient. The bar graph on the right side of the heatmap showed the frequency of the type of mutations in each gene. **B** Negative correlation of PNH clone size with frequency of uncommon mutations in PNH. Patients were divided into different groups (≤40%, 41–80%, >80%) according to their PNH clone size. Genes commonly mutated in and associated with a poor prognosis in aplastic anemia were uncommonly mutated in PNH. These mutated genes were lumped together. The frequency of these mutations declined in PNH as the size of PNH clone increased. **C** Relationship between gene mutations and sites of thrombosis in PNH. The mutation rates of SRRD and EGR4 in different subgroups of patients were shown in (**a**) and (**b**), respectively. The numbers on the columns represented the number of patients in each subgroup. Logistic regression revealed that mutation in SRRD was an independent risk factor for visceral thrombosis (*p* = 0.032) whereas mutation in EGR4 was an independent risk factor for myocardial infarction (*p* = 0.007). **D** Negative correlation between PNH clone size and frequency of mutations in thrombosis-related genes in PNH. This figure elucidates the PNH clone size calculated by FLAER negative granulocytes as well as the number of mutation in candidate genes concerning thrombosis in 13 patients with thrombosis events. The blue line is the fitted line of the correlation between clone sizes and number of mutations. Pearson correlation analysis revealed a negative correlation between clone sizes and number of mutations (*p* = 0.036).

Of the 178 genes frequently mutated in myeloid neoplasms (Supplementary Methods), 158 were mutated in our cohort (Fig. 1A). All 41 patients had mutations and 39 (95%) had multiple mutations. The average mutation load was 3.85 genes per patient (range, 1–9). As expected, *PIGA* was most commonly mutated and detected in 22 patients (53.7%). The types of *PIGA* mutations included truncation (*n* = 3), splicing-site mutations (*n* = 2), frame-shift deletion (*n* = 2), and missense mutation (*n* = 1); additional mutations located in intronic sites (*n* = 11) or in the 3ʹ untranslated region (*n* = 3). PIGT was mutated in one (2.4%) patient. Following *PIGA* gene, the most commonly mutated genes included *MAP3K4* and *CSMD1*, detected in 5 patients (12.2%) each. Genes mutated in 4 patients (9.8%) each included *NOTCH1, FANCD2, RUNX1T1, PEG3, DIS3, BCORL1* and *SETBP1*. Genes mutated in 3 patients (7.3%) each included *FANCG, RAD50, FANCA, CDH23, UMODL1, BRAF*, and *NCOR2*. In addition, 24 genes mutated in 2 patients and 43 in 1 patient each (Supplementary data).

We then examined whether these mutations correlated with clinical and laboratory parameters. We focused on 10 genes with the highest frequency of mutation, including *PIGA*, *BCORL1*, *RUNXT1*, *MAP3K*, *CSMD1*, *NOTCH1*, *FANCD2*, *PEG3*, *DIS3*, and *SETBP1* (Table 1). Patents with *PIGA* mutation had a larger PNH clone size than those without (90.6 ± 7.4% vs 64.4 ± 32.1%, *p* < 0.001), and were predominantly females (58.3% vs 3.6%, *p* < 0.001). Patients with *BCORL1* mutation were 20 years older (57 ± 19 vs 37 ± 13 year-old, *p* = 0.007). Patients with *RUNX1T1* mutation had a larger PNH clone of granulocytes (92.8 ± 5.4% vs 67.1 ± 31.3%, *p* < 0.001), a lower hemoglobin level (55.5 ± 13.3 g/L vs 83.7 ± 22.6 g/L, *p* = 0.020), and a higher level of unconjugated bilirubin (30.9 ± 16.2 μmol/L vs 15.6 ± 9.8 μmol/L, *p* = 0.008), suggestive of a higher tendency of hemolysis. No difference in clinical and laboratory parameters was observed between patients with and without 7 other most commonly mutated genes.

**Table 1:** Correlation of common mutations with clinical and laboratory features in PNH.

	Age (year old, SD)	Sex (male %)	Classical PNH (%)	Patients with thrombosis (%)	FLAER- (%, SD)	UCB (μmol/L, SD)	HGB (g/L, SD)	RET (10^9^/L, SD)	LDH (U/L, SD)
*PIGA*									
Mutated	39 (12)	13***	50	50	90.6 (7.3)***	15.8 (10.0)	79.9 (15.1)	21.0 (12.6)	1311 (899)
Unmutated	39 (14)	85***	45	27	64.4 (32.1)***	17.4 (11.7)	81.2 (25.1)	30.5 (63.7)	1159 (734)
*MAP3K4*									
Mutated	34 (14)	60	20	20	72.4 (35.4)	14.2 (13.2)	72.8 (18.8)	10.8 (10.8)	1189 (1057)
Unmutated	40 (15)	72	50	33	68.2 (30.6)	17.5 (11.1)	82.1 (23.9)	30.8 (60.8)	1189 (729)
*CSMD1*									
Mutated	37 (19)	60	20	0	56.6 (42.9)	12.6 (8.8)	79.8 (27.1)	9.9 (6.8)	779 (565)
Unmutated	40 (14)	72	50	33	71.4 (29.0)	17.7 (11.5)	81.1 (23.2)	31.5 (61.6)	1246 (771)
*NOTCH1*									
Mutated	41 (9)	50	75	50	63.0 (37.4)	9.8 (4.8)	94.5 (26.1)	15.0 (8.4)	787 (404)
Unmutated	39 (15)	73	43	30	70.3 (30.5)	17.8 (11.5)	79.5 (22.9)	30.3 (61.0)	1232 (779)
*FANCD2*									
Mutated	37 (16)	20	25	25	67.7 (35.6)	11.1 (8.9)	72.5 (20.4)	18.8 (19.0)	1165 (1323)
Unmutated	40 (14)	76	49	32	69.8 (30.7)	17.7 (11.4)	81.9 (23.7)	29.9 (60.9)	1191 (703)
*RUNX1T1*									
Mutated	36 (4)	100	75	25	92.8 (5.4)***	30.9 (16.2)**	55.5 (13.3)*	31.6 (30.4)	1301 (565)
Unmutated	40 (15)	68	43	32	67.0 (31.3)***	15.6 (9.8)**	83.7 (22.6)*	28.4 (60.5)	1177 (783)
*PEG3*									
Mutated	50 (22)	75	0	25	59.5 (33.7)	16.7 (11.7)	81.3 (23.0)	54.6 (85.4)	1355 (1141)
Unmutated	38 (13)	70	51	32	70.6 (30.7)	17.1 (11.4)	81.0 (23.7)	25.8 (54.9)	1171 (727)
*DIS3*									
Mutated	40 (19)	100	75	25	75.7 (15.0)	13.9 (6.7)	92.5 (34.3)	13.7 (10.1)	970 (502)
Unmutated	39 (14)	68	43	32	68.9 (32.0)	17.4 (11.7)	79.7 (22.2)	30.5 (60.9)	1212 (783)
*BCORL1*									
Mutated	57 (19)**	50	0	25	53.2 (28.6)	17.9 (11.1)	65.3 (17.3)	53.8 (85.4)	1316 (1069)
Unmutated	37 (13)**	73	51	32	71.3 (30.8)	17.0 (11.4)	82.7 (23.5)	25.9 (55.0)	1175 (737)
*SETBP1*									
Mutated	47 (13)	100	75	0	69.0 (35.7)	16.9 (8.9)	76.3 (28.2)	13.1 (10.4)	1050 (639)
Unmutated	38 (15)	68	43	35	69.6 (30.7)	17.1 (11.6)	81.5 (23.2)	30.6 (60.9)	1204 (777)

Measurement data were presented as average (standard deviation).

*FLAER-* proportion of fluorescent aerolysin-negative granulocytes, *UCB* unconjugated bilirubin, *HGB* hemoglobin, *RET* reticulocyte count, *LDH* lactic dehydrogenase.

*p < 0.05, **p < 0.01, ***p < 0.001.

Since the incidence of commonly mutated genes associated with a worse outcome in AA was generally lower in PNH patients except for *CSMD1* (12.2%): 2.4% for *DNMT3A*, 2.4% for *RUNX1*, 4.9% for *JAK2*, and 4.9% for *JAK3*, we lumped patients with these mutations together. As one group, patients carrying these mutations had a smaller PNH clone of granulocytes (48.3 ± 35.9 vs 75.5 ± 26.8, *p* = 0.017), a lower level of LDH (731.9 ± 443.2 vs 1317.2 ± 784.6, *p* = 0.008), and a lower level of UCB (11.2 ± 6.7 vs 18.7 ± 11.8, *p* = 0.022), suggestive of a lower tendency of hemolysis. No difference in other clinical and laboratory parameters was observed between patients with and patients without these uncommonly mutated genes (Supplementary Table S2).

To further explore the correlation between the PNH clone size and the mutational frequency of genes indicating worse outcome in AA, we divided the patients into 3 groups according to their clone size: ≤40% (*n* = 9), 41–80% (*n* = 10), and >80% (*n* = 22). The mutational frequency of those genes was 44% (4/9) in patients with PNH clone size ≤40%, 30% (3/10) in those with clone size of 41–80%, and 9% (2/22) in patients with clone size >80%. Logistic regression analysis indicated that the mutational frequency declined with the increase of PNH clone size (*p* = 0.026) (Fig. 1B).

Next we investigated the potential role of mutations in clonal expansion in sorted CD59+ and CD59- cells of peripheral blood from 6 patients with a relatively large PNH clone. Overall the mutation frequencies were similar in CD59- and CD59+ population, except for *PIGA* mutation, which was detected only in CD59- population. We then searched for those associated with cell proliferation according to the criteria in Supplementary Methods and compared them between sorted CD59- and CD59+ cells. Of the 723 genes associated with cell proliferation, 210 were found mutated in the 6 patients. The mutational loads of those 210 genes were 46.7 (range, 41–53) and 48 (range, 42–52) in the CD59 + and CD59- cells, respectively. Mutated genes unique in CD59- cells included *MUC16, NCOR2, PTPN11, CIC, MAML2, BCR, RGPD3, ARID1A, KMT2C, MSH2, NCOR2*, and *TCL1A*, and mutations found in CD59 + cells only included *ROBO2, SF3B1, H2B3, and BCR*. Overall, there was a strong trend toward more cell proliferation or clone expansion associated mutations in CD59- cells than in CD59 + cells (*p* = 0.062) (Supplementary Table S3).

Then we examined the potential role of mutations in thrombosis. A total of 55 thrombosis-related genes (Supplementary Table S4) were selected as the candidate genes according to the criteria in Supplementary Methods. No difference in mutation rate was found in those candidate genes between CD59 + and CD59- cells. In addition, mutation in *SRRD gene* was more common in patients with visceral thrombosis than those with thrombosis in other sites (80% vs 12.5%, *p* = 0.032), whereas *EGR4* mutation was more common in patients with myocardial infarction (100% vs 11.1%, *p* = 0.007) (Fig. 1C). In patients with thrombosis, the number of those candidate mutations was negatively correlated with the PNH clone size (*p* = 0.036, *R*^2^ = 0.341) (Fig. 1D), i.e. those with relatively smaller PNH clone sizes tended to have more thrombosis-related mutations, especially mutations of *PADI1* (*p* = 0.001), *SLC2A9* (*p* = 0.002) and *TCF3* (*p* = 0.011) gene.

In summary, by whole-exome sequencing 10 most frequently mutated genes in PNH included *PIGA, BCORL1, RUNX1T1, MAP3K4*, *CSMD1*, *NOTCH1, FANCD2, PEG3, DIS3*, and *SETBP1. PIGA* mutation was associated with a larger PNH clone size and female sex, *BCORL1* mutation was associated with a younger age, and *RUNX1T1* mutation correlated with a larger PNH clone size, a lower hemoglobin level, and a higher level of unconjugated bilirubin. Mutations indicating an unfavorable outcome in AA were uncommon in PNH and as one group associated with a smaller PNH clone size, a lower level of LDH, and a lower level of unconjugated bilirubin. CD59- fraction tended to have more mutations in proliferation-related genes compared with CD59 + fraction. Thrombosis in different sites demonstrated different gene mutations. *SRRD* mutation was associated with visceral thrombosis and *EGR4* mutation was associated with myocardial infarction. For the first time, we demonstrated the clinical significance of mutation profile in PNH, particularly, in PNH clonal expansion and thrombosis.

## Supplementary information

Supplementary data: uncommon mutations in PNH

SUPPLEMENTARY METHODS

Table S1. Clinical and laboratory features of patients with vs without thrombosis

Table S2. Correlation of uncommon mutations with clinical and laboratory features in PNH

Table S3 Cell proliferation related genes in CD 59- and CD59+ cells

Table S4. Candidate genes involved in thrombosis in PNH patients
