# Author Correction: Mutational landscape and its clinical significance in paroxysmal nocturnal hemoglobinuria

**DOI:** 10.1038/s41408-021-00476-6

**Published:** 2021-05-06

**Authors:** Fangfei Chen, Shimin Hu, Jing Ruan, Miao Chen, Bing Han

**Affiliations:** 1 Department of Hematology, Peking Union Medical College Hospital, Peking Union Medical College and Chinese Academy of Medical Sciences, Beijing, China; 2 Division of Gastroenterology, The First Affiliated Hospital, Sun Yat-sen University, Guangzhou, China; 3 Department of Hematopathology, The University of Texas MD Anderson Cancer Center, Houston, TX USA

**Keywords:** Disease genetics, Anaemia, Genetics research, Disease genetics

Correction to: *Blood Cancer Journal*

10.1038/s41408-021-00451-1 published online 16 March 2021

When examining our submitted paper, we found 2 mistakes that need to be changed.

1. Author affiliations:

The affiliations of the first author Fangfei Chen should be (1) Department of Hematology, Peking Union Medical College Hospital, Peking Union Medical College and Chinese Academy of Medical Sciences, Beijing, China. (2) Division of Gastroenterology, The First Affiliated Hospital, Sun Yat-sen University, Guangzhou, China.

2. Funding:

One funding had been missed: The National Key Research and Development Program of China (2016YFC0901500).

