# Supplementary material for: Mutational landscape and its clinical significance in paroxysmal nocturnal hemoglobinuria
Source: Blood Cancer J. 2021 Mar 16;11(3):58. doi: 10.1038/s41408-021-00451-1 (PMC7966366; doi:10.1038/s41408-021-00451-1)
Supplement: Supplementary file 1 — Supplementary data: uncommon mutations in PNH [file 41408_2021_451_MOESM1_ESM.docx]

**Supplementary data: uncommon mutations in PNH**

**24 genes mutated in 2 patients:**

*TCF4, SRP72, SBDS, RPS10, RINT1, NUP98, MPL, MN1, JARID2, JAK3, JAK2, HMGA2, GNAS, GATA1, DIDO1, CTNNA1, CEBPA, CD101, CBLC, CBLB, ACTRS, ABCA12, POT1, PKP3*

**43 genes mutated in one patient:**

*RUNX1, RAD21, KDM5A, TPP1, TINF2, TET2, TET1, SF3B1, RPS19, RBBP4, PRPF40B, PRF1, PHF12, OCA2, NLRP1, MMD2, MET, MECOM, LAMB4, KIT, KDM2B, IRF1, INVS, IDH1, HIPK2, FLT3, FBXW7, FANCE, ETV6, EP300, ELF1, EGFR, EED, DNMT3A, DDX41, CUX1, CSF1R, CHGA, CDKN2A, CDKN1B, CDH1, CDAN1, ATM*
