# Supplementary material for: Mutational landscape and its clinical significance in paroxysmal nocturnal hemoglobinuria
Source: Blood Cancer J. 2021 Mar 16;11(3):58. doi: 10.1038/s41408-021-00451-1 (PMC7966366; doi:10.1038/s41408-021-00451-1)
Supplement: Supplementary file 2 — SUPPLEMENTARY METHODS [file 41408_2021_451_MOESM2_ESM.docx]

## Supplementary Methods

## Patient selection

Patients with PNH newly diagnosed at Peking Union Medical College Hospital from April 2017 through April 2019 according to the previously established criteria were included in the study (1). These patients were further stratified into classical PNH or PNH/AA subgroups according to the criteria established by the International PNH Interest Group (2). Their clinical information and laboratory results were recorded. The study was approved by the Institutional Review Board of Peking Union Medical College.

**Detection of PNH clones by flow cytometry**

Flow cytometry with fluorescent aerolysin (FLAER) was used to determine the PNH clone. The PNH clone size was calculated based on the proportion of FLAER-negative granulocytes detected by flow cytometry (2).

**Cell sorting and separation of CD59- and CD59+ leukocytes**

Extraction of leukocytes from peripheral blood specimens of 6 patients was conducted after lysis of erythrocytes. Cell sorting was performed to separate CD59- and CD59+ leukocytes as described previously (3). Briefly, cells were sorted using anti-CD59 PE (BD PharMingen, USA), anti-PE microbeads (Miltenyi Biotec, Germany) and immunomagnetic separation column (Miltenyi Biotec) according to the Manufacturer’s instructions. The purity of CD59- and CD59+ fractions were confirmed by flow cytometry (BD Biosciences).

**Whole-exome sequencing**

DNA was extracted from 4 ml of peripheral blood from each patient or magnetically sorted CD59- and CD59+ cells from 6 patients using PureLink™ Genomic DNA Mini Kit (Invitrogen) according to the Manufacturer’s instructions. Agarose gel electrophoresis was employed to ensure the concentration, purity and integrity of DNA. WES was performed as follows: the library construction was performed using Agilent SureSelect Human All Exon V6 kit, the quality of library was confirmed by Qubit 2.0, NGS3K/Caliper and qPCR, and high-throughput sequencing was performed using Illumina HiSeq2500 instrument (Illumina, Inc.). Bioinformatic analysis was conducted using the Genome Reference Consortium Human Genome Build 37 (hg19/GRCh37; [ftp://ftp.ncbi.nlm.nih.gov/genomes/H_sapiens](https://ftp.ncbi.nlm.nih.gov/genomes/H_sapiens)) as the reference genome. The average depth of sequencing was 127×.

**Classification criteria for mutations**

The pipeline analysis of sequencing data was as follows: (1) Frequency filtration: SNPs with frequency lower than 1% were retained according to the database of 1000-genome, ESP6500 and gnomAD. (2) Site filtration: Nonsynonymous mutations were retained in coding area, splicing sites or highly conserved regions. (3) Function filtration: Mutations were retained which may have impact on splicing by dbscSNV, or be pathogenic in more than half of the following software: SIFT, Polyphen, MutationTaster, and CADD (4, 5). Mutations are classified into 5 groups according to the standard guidelines for the interpretation of sequence variants proposed by the American College of Medical Genetics and Genomics: pathogenic, likely pathogenic, uncertain significance, likely benign and benign (6).

**Analysis of myeloid cancer-related gene mutations and their clinical relevance**

Mutations in 178 candidate genes commonly mutated in myeloid neoplasms were analyzed in our sample (7, 8), as well as their correlation with the clinical manifestations, such as sex, age, blood cell count, level of hemoglobin, lactate dehydrogenase, unconjugated bilirubin, and PNH clone size. The distribution of these gene mutations in the sorted CD59+ and CD59- fraction was analyzed as well.

**Mutations associated with clonal expansion of PNH**

Among the pathogenic, likely pathogenic and uncertain significance mutations, we searched for those associated with cell proliferation according to the Cancer Gene Census (<https://cancer.sanger.ac.uk/census>) database in Catalogue of Somatic Mutations in Cancer (9). A total of 723 genes were selected as candidate genes and compared between sorted CD59- and CD59+ cells from each patient.

**Mutations associated with thrombosis**

Candidate genes potentially associated with thrombosis were selected according to the following criteria. 1) mutations classified as pathogenic, likely pathogenic or uncertain significance according to ACMG. 2) genes with potential involvement in coagulation according to Phenolyzer (http://phenolyzer.usc.edu) (10). 3) genes with significantly higher mutational rates in thrombotic patients compared with non- thrombotic patients.

**Statistical analysis**

SPSS16.0 (SPSS Inc., Chicago, IL, USA) was used for statistical analysis. A p<0.05 was considered as statistically significant. T-test (for normal distribution sample) or Mann-Whitney U test (for abnormal distribution sample) was conducted for the comparison of measurement data. Chi-square test was used for the comparison between categorical data. Multiple stepwise linear regression and logistic regression were used to analyze the correlation between mutations and clinical features. Kruskal-Wallis test was performed for the comparison of mutational distribution in multiple groups. Wilcoxon signed rank test was used to analyze the difference of mutations between paired CD59- and CD59+ cell fractions. Pearson correlation analysis was used to examine the relation between PNH clone size and number of mutations in thrombosis-candidate genes.

Reference

1. Pu JJ, Brodsky RA. Paroxysmal Nocturnal Hemoglobinuria from Bench to Bedside. Clinical and Translational Science. 2011;4(3):219-24.

2. Parker C, et al. Diagnosis and management of paroxysmal nocturnal hemoglobinuria. Blood. 2005;106(12):3699-709.

3. Shen W, et al. Deep sequencing reveals stepwise mutation acquisition in paroxysmal nocturnal hemoglobinuria. The Journal of clinical investigation. 2014;124(10):4529-38.

4. Ng PC, Henikoff S. Predicting deleterious amino acid substitutions. Genome research. 2001;11(5):863-74.

5. Choi Y, Sims GE, Murphy S, Miller JR, Chan AP. Predicting the functional effect of amino acid substitutions and indels. PLoS One. 2012;7(10):e46688.

6. Richards S, et al. Standards and guidelines for the interpretation of sequence variants: a joint consensus recommendation of the American College of Medical Genetics and Genomics and the Association for Molecular Pathology. Genetics in medicine : official journal of the American College of Medical Genetics. 2015;17(5):405-24.

7. Yoshizato T, et al. Somatic Mutations and Clonal Hematopoiesis in Aplastic Anemia. New England Journal of Medicine. 2015;373(1):35-47.

8. Papaemmanuil E, et al. Clinical and biological implications of driver mutations in myelodysplastic syndromes. Blood. 2013;122(22):3616-27; quiz 99.

9. Tate JG, et al. COSMIC: the Catalogue Of Somatic Mutations In Cancer. Nucleic acids research. 2019;47(D1):D941-d7.

10. Yang H, Robinson PN, Wang K. Phenolyzer: phenotype-based prioritization of candidate genes for human diseases. Nature methods. 2015;12(9):841-3.
