# Supplementary material for: Mutational landscape and its clinical significance in paroxysmal nocturnal hemoglobinuria
Source: Blood Cancer J. 2021 Mar 16;11(3):58. doi: 10.1038/s41408-021-00451-1 (PMC7966366; doi:10.1038/s41408-021-00451-1)
Supplement: Supplementary file 3 — Table S1. Clinical and laboratory features of patients with vs without thrombosis [file 41408_2021_451_MOESM3_ESM.docx]

**Table S1. Clinical and laboratory features of patients with vs without thrombosis**

| Parameters | With thrombosis | Without thrombosis | p |
| --- | --- | --- | --- |
| Number | 13 (31.7%) | 28 (68.3%) | - |
| Sex (Male/Female) | 10/3 | 19/9 | 0.822 |
| Age | 34 (25-58) | 38 (15-72) | 0.817 |
| Clinical classification (PNH-AA/classical PNH) | 6/7 | 16/12 | 0.511 |
| WBC (10^9^/L) | 4.20 (1.70-6.30) | 3.55 (1.50-10.90) | 0.853 |
| NEU (10^9^/L) | 1.18 (0.18-3.14) | 1.24 (0.11-3.46) | 0.603 |
| RBC (10^12^/L) | 2.80 (1.60-4.67) | 2.47 (1.20-3.54) | 0.108 |
| HGB (g/L) | 86.0 (51.0-140.0) | 76.5 (36.0-126.0) | 0.148 |
| PLT (10^9^/L) | 111.0 (21.0-349.0) | 92.5 (11.0-348.0) | 0.379 |
| LDH (U/L) | 943.0 (183.0-3114.0) | 1050.0 (175.0-2674.0) | 0.991 |
| UCB (μmol/L) | 11.3 (4.5-36.4) | 12.9 (4.6-50.2) | 0.729 |
| D-dimer (mg/L) | 2.96 (0.26-5.57) | 0.42 (0.15-3.50) | 0.007 |
| PNH clone size (%) | 83.0 (18.0-95.0) | 83.0 (10.0-98.0) | 0.730 |

PNH, paroxysmal nocturnal hemoglobinuria; AA, aplastic anemia; RBC, red blood cell; WBC, white blood cell; NEU, neutrophil; HGB, hemoglobin; PLT, platelet; LDH, lactic dehydrogenase; UCB, unconjugated bilirubin; PNH clone size was calculated by FLAER- granulocytes.
