# Supplementary material for: Mutational landscape and its clinical significance in paroxysmal nocturnal hemoglobinuria
Source: Blood Cancer J. 2021 Mar 16;11(3):58. doi: 10.1038/s41408-021-00451-1 (PMC7966366; doi:10.1038/s41408-021-00451-1)
Supplement: Supplementary file 4 — Table S2. Correlation of uncommon mutations with clinical and laboratory features in PNH [file 41408_2021_451_MOESM4_ESM.docx]

**Table S2. Correlation of uncommon mutations with clinical and laboratory features in PNH**

|  | Mutated | Unmutated | P-value |
| --- | --- | --- | --- |
| Classical PNH (%) | 22 | 53 | 0.206 |
| Patients with thrombosis (%) | 22 | 34 | 0.774 |
| Age (year, SD) | 40.9 (18.2) | 38.9 (13.5) | 0.717 |
| FLAER- (%, SD) | 48.3 (35.9) | 75.5 (26.8) | 0.017 |
| UCB (μmol/L, SD) | 11.2 (6.7) | 18.7 (11.8) | 0.022 |
| HGB (g/L, SD) | 73.0 (24.5) | 83.2 (22.9) | 0.251 |
| RET (10^9^/L, SD) | 10.1 (8.3) | 34.4 (65.1) | 0.276 |
| LDH (U/L, SD) | 731.9 (443.2) | 1317.2 (784.6) | 0.008 |

Data were presented as average (standard variation). Uncommon mutations are a group of mutations indicating worse outcome in AA, including *DNMT3A, RUNX1, JAK2, JAK3* and *CSMD1*.

FLAER-, proportion of fluorescent aerolysin-negative granulocytes; UCB, unconjugated bilirubin; HGB, hemoglobin; RET, reticulocyte count; LDH, lactate dehydrogenase.
