# Supplementary material for: Mutational landscape and its clinical significance in paroxysmal nocturnal hemoglobinuria
Source: Blood Cancer J. 2021 Mar 16;11(3):58. doi: 10.1038/s41408-021-00451-1 (PMC7966366; doi:10.1038/s41408-021-00451-1)
Supplement: Supplementary file 5 — Table S3 Cell proliferation related genes in CD 59- and CD59+ cells [file 41408_2021_451_MOESM5_ESM.docx]

**Table S3 Cell proliferation related genes in CD 59- and CD59+ cells**

| Patient # | PNH clone size (%) | Mutation load | | | mutations only in CD59+ or CD59- cells | |
| --- | --- | --- | --- | --- | --- | --- |
|  |  | total | CD59- | CD59+ | CD59- cells | CD59+ cells |
| 1 | 98 | 50 | 50 | 48 | *MUC16* |  |
|  |  |  |  |  | *NCOR2* |  |
| 2 | 47 | 50 | 50 | 49 | *PTPN11* |  |
| 3 | 93 | 43 | 43 | 41 | *CIC* |  |
|  |  |  |  |  | *MAML2* |  |
| 4 | 98 | 43 | 42 | 41 | *BCR* | *ROBO2* |
|  |  |  |  |  | *RGPD3* |  |
| 5 | 70 | 55 | 52 | 53 | *ARID1A* | *SF3B1* |
|  |  |  |  |  | *KMT2C* | *SH2B3* |
|  |  |  |  |  |  | *BCR* |
| 6 | 84 | 51 | 51 | 48 | *MSH2* |  |
|  |  |  |  |  | *NCOR2* |  |
|  |  |  |  |  | *TCL1A* |  |
