# Supplementary material for: Mutational landscape and its clinical significance in paroxysmal nocturnal hemoglobinuria
Source: Blood Cancer J. 2021 Mar 16;11(3):58. doi: 10.1038/s41408-021-00451-1 (PMC7966366; doi:10.1038/s41408-021-00451-1)
Supplement: Supplementary file 6 — Table S4. Candidate genes involved in thrombosis in PNH patients [file 41408_2021_451_MOESM6_ESM.docx]

**Table S4. Candidate genes involved in thrombosis in PNH patients**

| Gene | Number of mutation in group with thrombosis  (mutation rate) | Number of mutation in group without thrombosis  (mutation rate) | p value | RR |
| --- | --- | --- | --- | --- |
| Signaling | | | | |
| *SGK2* | 9 (69.2%) | 10 (35.7%) | 0.045 | 2.61 |
| *CHRNG* | 7 (53.8%) | 3 (10.7%) | 0.009 | 3.62 |
| *IL11RA* | 7 (53.8%) | 5 (17.9%) | 0.047 | 2.82 |
| *OR51L1* | 4 (30.8%) | 0 | 0.012 | 4.11 |
| *LRRFIP1* | 3 (23.1%) | 0 | 0.027 | 3.80 |
| *NR4A1* | 3 (23.1%) | 0 | 0.027 | 3.80 |
| *PRMT7* | 3 (23.1%) | 0 | 0.027 | 3.80 |
| *OR6C4* | 3 (23.1%) | 0 | 0.027 | 3.80 |
| *EPHB1* | 3 (23.1%) | 0 | 0.027 | 3.80 |
| *OR4C6* | 3 (23.1%) | 0 | 0.027 | 3.80 |
| *OR5L1* | 3 (23.1%) | 0 | 0.027 | 3.80 |
| *DYNLRB1* | 3 (23.1%) | 0 | 0.027 | 3.80 |
| *PPP2R5B* | 3 (23.1%) | 0 | 0.027 | 3.80 |
| Transcription | | | | |
| *MED15* | 7 (53.8%) | 3 (10.7%) | 0.009 | 3.62 |
| *ESF1* | 6 (46.2%) | 3 (10.7%) | 0.032 | 3.05 |
| *TCF3* | 6 (46.2%) | 2 (7.1%) | 0.012 | 3.54 |
| *FOXP3* | 5 (38.5%) | 1 (3.6%) | 0.014 | 3.65 |
| *EGR4* | 5 (38.5%) | 1 (3.6%) | 0.014 | 3.65 |
| *SIX4* | 4 (30.8%) | 0 | 0.012 | 4.11 |
| *DDX1* | 3 (23.1%) | 0 | 0.027 | 3.80 |
| *DLX2* | 3 (23.1%) | 0 | 0.027 | 3.80 |
| *ZNF215* | 3 (23.1%) | 0 | 0.027 | 3.80 |
| *COQ7* | 3 (23.1%) | 0 | 0.027 | 3.80 |
| *IKZF4* | 3 (23.1%) | 0 | 0.027 | 3.80 |
| Transportation | | | | |
| *SEC16A* | 8 (61.5%) | 6 (21.4%) | 0.03 | 3.09 |
| *KCNH6* | 5 (38.5%) | 1 (3.6%) | 0.014 | 3.65 |
| *SLC28A3* | 5 (38.5%) | 0 | 0.003 | 3.50 |
| *SLC25A10* | 5 (38.5%) | 0 | 0.003 | 3.50 |
| *SLC2A13* | 4 (30.8%) | 0 | 0.012 | 4.11 |
| *SLC44A3* | 3 (23.1%) | 0 | 0.027 | 3.80 |
| *DAB2* | 3 (23.1%) | 0 | 0.027 | 3.80 |
| *SLC2A9* | 3 (23.1%) | 0 | 0.027 | 3.80 |
| Immune reaction | | | | |
| *LRBA* | 7 (53.8%) | 4 (14.3%) | 0.023 | 3.18 |
| *PRB3* | 6 (46.2%) | 3 (10.7%) | 0.032 | 3.05 |
| *LEAP2* | 4 (30.8%) | 0 | 0.012 | 4.11 |
| *ELMO2* | 4 (30.8%) | 0 | 0.012 | 4.11 |
| *IFIT2* | 3 (23.1%) | 0 | 0.027 | 3.80 |
| Ubiquitination | | | | |
| *HECTD1* | 7 (53.8%) | 4 (14.3%) | 0.023 | 3.18 |
| *KLHL28* | 5 (38.5%) | 2 (7.1%) | 0.042 | 3.04 |
| *UBOX5* | 3 (23.1%) | 0 | 0.027 | 3.80 |
| *DERL3* | 3 (23.1%) | 0 | 0.027 | 3.80 |
| Calcium binding | | | | |
| *MEGF6* | 8 (61.5%) | 6 (21.4%) | 0.03 | 3.09 |
| *ENPP2* | 5 (38.5%) | 2 (7.1%) | 0.042 | 3.04 |
| *PADI3* | 4 (30.8%) | 0 | 0.012 | 4.11 |
| *PADI1* | 3 (23.1%) | 0 | 0.027 | 3.80 |
| Metabolism | | | | |
| *MRI1* | 5 (38.5%) | 2 (7.1%) | 0.042 | 3.04 |
| *TMEM5* | 3 (23.1%) | 0 | 0.027 | 3.80 |
| *CYP2B6* | 3 (23.1%) | 0 | 0.027 | 3.80 |
| Others | | | | |
| *COL6A3* | 8 (61.5%) | 5 (17.9%) | 0.015 | 3.45 |
| *NLRP5* | 6 (46.2%) | 2 (7.1%) | 0.012 | 3.54 |
| *SRRD* | 5 (38.5%) | 1 (3.6%) | 0.014 | 3.65 |
| *MYLK3* | 4 (30.8%) | 0 | 0.012 | 4.11 |
| *RTEL1* | 3 (23.1%) | 0 | 0.027 | 3.80 |
| *COL9A1* | 3 (23.1%) | 0 | 0.027 | 3.80 |
| *LYPD3* | 3 (23.1%) | 0 | 0.027 | 3.80 |
